# Low HER2 expression in normal breast epithelium enables dedifferentiation and malignant transformation via chromatin opening

**DOI:** 10.1242/dmm.049894

**Published:** 2023-02-01

**Authors:** Ateequllah Hayat, Edward P. Carter, Hamish W. King, Aysegul Ors, Aaron Doe, Saul A. Teijeiro, Sarah Charrot, Susana Godinho, Pedro Cutillas, Hisham Mohammed, Richard P. Grose, Gabriella Ficz

**Affiliations:** ^1^ Institute of Medical and Biomedical Education, St George’s, University of London, Cranmer Terrace, Tooting, London SW17 0RE, UK; ^2^ Centre for Haemato-Oncology, Barts Cancer Institute, Queen Mary University of London, London EC1M 6BQ, UK; ^3^ Centre for Tumour Biology, Barts Cancer Institute, Queen Mary University of London, London EC1M 6BQ, UK; ^4^ Epigenetics and Development Division, Walter and Eliza Hall Institute of Medical Research, Royal Parade, Parkville, VIC 3052, Australia; ^5^ Knight Cancer Institute, Oregon Health and Science University, 3181 S.W. Sam Jackson Park Road, Portland, OR 97239-3098, USA; ^6^ Centre for Molecular Oncology, Barts Cancer Institute, Queen Mary University of London, London EC1M 6BQ, UK

**Keywords:** Breast, Cancer, Chromatin, Epigenetics, *In vitro*, Stem

## Abstract

Overexpression of the HER2 protein in breast cancer patients is a predictor of poor prognosis and resistance to therapies. We used an inducible breast cancer transformation system that allows investigation of early molecular changes. HER2 overexpression to similar levels as those observed in a subtype of HER2-positive breast cancer patients induced transformation of MCF10A cells and resulted in gross morphological changes, increased anchorage-independent growth of cells, and altered the transcriptional programme of genes associated with oncogenic transformation. Global phosphoproteomic analysis during HER2 induction predominantly detected an increase in protein phosphorylation. Intriguingly, this correlated with chromatin opening, as measured by ATAC-seq on acini isolated from 3D cell culture. HER2 overexpression resulted in opening of many distal regulatory regions and promoted reprogramming-associated heterogeneity. We found that a subset of cells acquired a dedifferentiated breast stem-like phenotype, making them likely candidates for malignant transformation. Our data show that this population of cells, which counterintuitively enriches for relatively low HER2 protein abundance and increased chromatin accessibility, possesses transformational drive, resulting in increased anchorage-independent growth *in vitro* compared to cells not displaying a stem-like phenotype.

## Teaser

**Summary:** Low HER2 expression promotes *in vitro* transformation in a 3D breast cancer model through widespread increase in chromatin accessibility and the acquisition of stem-like phenotype.

## INTRODUCTION

Metastasis is the main cause of cancer death, but understanding the root cause of malignant transformation remains poorly understood. Many questions remain unanswered as to what triggers cancer formation beyond DNA mutations in pre-cancerous tissue (Ciccarelli and DeGregori, 2020). Perturbed signalling due to dysregulated phosphorylation of oncogenic proteins is known to alter pathway activity and contributes to cellular transformation (Sever and Brugge, 2015; Hanahan and Weinberg, 2011). Similarly, cell identity and cellular plasticity are phenotypic outcomes of the signalling and epigenetic information in both healthy and disease states (Wainwright and Scaffidi, 2017). Therefore, understanding how an altered signalling environment affects the epigenome and shifts cellular states is crucial in furthering our understanding of cancer formation. Integrating systematic analyses of phosphorylation sites (phosphosites) from global phosphoproteomics data with DNA/RNA-sequencing data helps to better understand the functional significance of the signalling effects on chromatin changes. Phenotypic changes that occur during cancer development are driven by changes in the gene expression patterns, which are themselves governed by regulatory states encoded within the nucleoprotein structure of chromatin (Voss and Hager, 2014). The alterations in chromatin structure that lead to differential accessibility to transcription factor binding have been identified as perhaps some of the most relevant genomic characteristics correlated with biological activity at a specific locus (Thurman et al., 2012). Nevertheless, the specific regulatory changes driving the transition from normal to transformed cells remain largely unknown.

HER2 (ERBB2)-positive breast cancer accounts for ∼20% of all breast cancers (Wang and Xu, 2019). The ability of HER2-positive breast cancer cells to leave the primary tumour site and establish inoperable metastasis is a major cause of death and a serious impediment to successful therapy. Molecular analysis of HER2-positive breast cancer progression is limited by the inability to characterise and catalogue early changes at the onset of transformation. Conventional *in vitro* models (Pradeep et al., 2012; Gangadhara et al., 2016) can recapitulate the genetics, morphology, therapeutic response and highly transformative nature of the disease. However, they do not allow for the fine tuning and temporal control required to fully assess cellular events leading up to malignant transformation. To overcome this issue, we developed an inducible *in vitro* model of human breast cancer to investigate the mechanisms that drive early transformational changes in HER2-positive breast cancer. The strength of an inducible system lies in that it can recapitulate key transitional states in cancer progression in a controlled manner, permitting isolation of cancer-like cells at defined stages of transformation to catalogue early tumour-promoting changes.

Here, we analysed HER2 protein overexpression in a normal diploid, oestrogen- and progesterone-negative breast epithelial cell line, MCF10A (Qu et al., 2015), to identify global cell signalling and chromatin accessibility changes in the first few hours and days of cellular transformation. In particular, we explored how cell signalling interacts with chromatin to induce transformation as a result of HER2 pathway activation.

## RESULTS

### Conditional HER2 overexpression promotes *in vitro* transformation

**Fig. 1.**
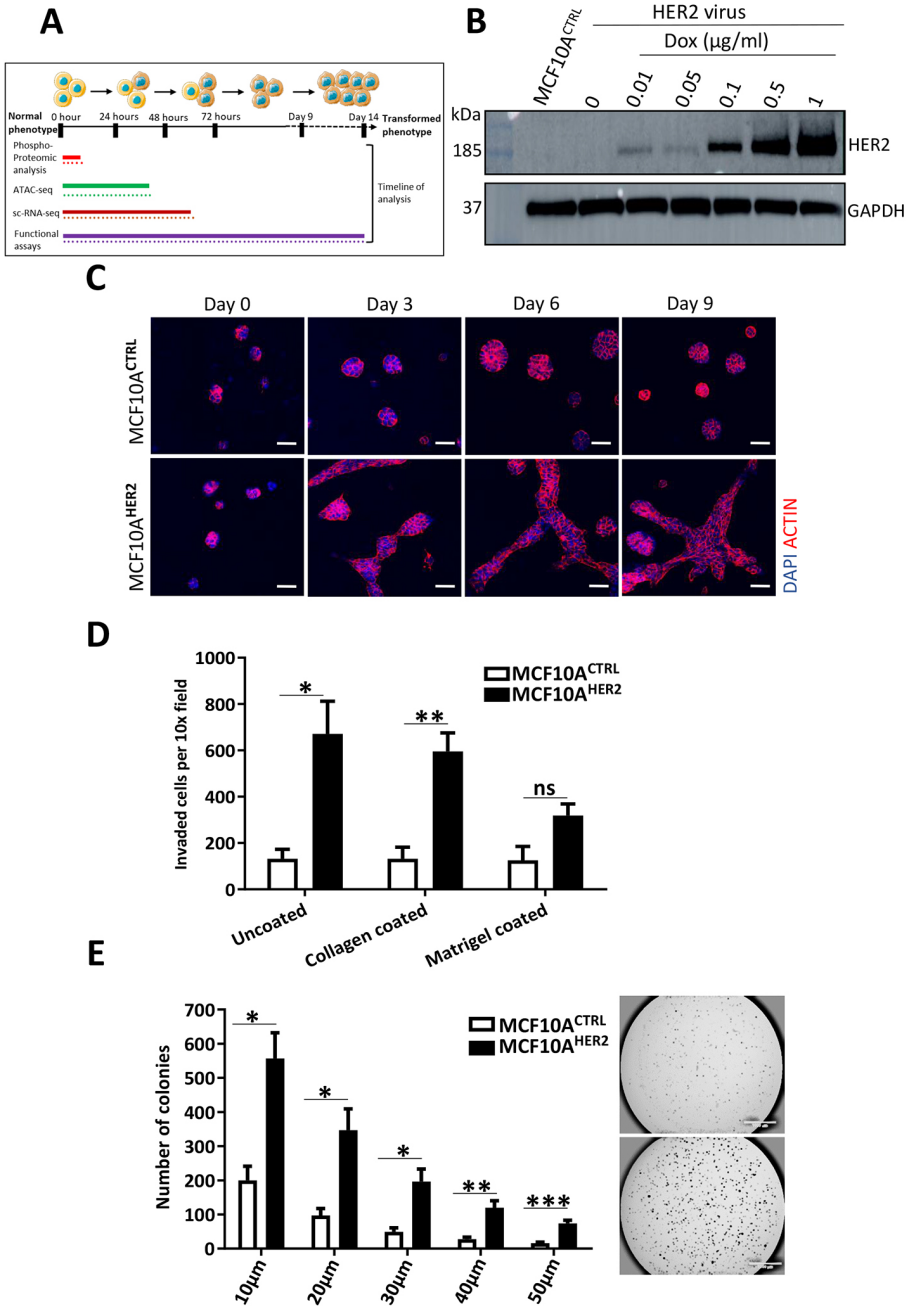
**HER2 protein overexpression is sufficient to induce *in vitro* transformation.** (A) Schematic of multi-omic analysis and soft functional assays performed with their respective timelines as MCF10A cells undergo *in vitro* transformation. ATAC-seq, assay of transposase-accessible chromatin using sequencing; scRNA-seq, single-cell RNA sequencing. (B) HER2 protein expression analysis by western blotting in MCF10A cells infected with inducible HER2 lentiviral particles and cultured in various concentration of doxycycline for 24 h. GAPDH was used as a loading control. *n*=2. (C) MCF10A^HER2^ and control cells were cultured in 3D over 9 days. Control cells formed spherical acini, which increased in size over time. MCF10A^HER2^ cells formed flat projecting cells of complex masses, typical of transformed cells. Images captured by a confocal, LSM 510 microscope. Scale bars: 50 µm. *n*=3. (D) Cell migration and invasion was analysed through the 8 µm pores of transwell membranes over a 16 h period of chemotactic migration towards full serum medium. The ability for cell invasion was measured in collagen or Matrigel-coated transwells. Migration ability was measured in using uncoated wells. Statistical significance was calculated using unpaired two-tailed Student's *t*-test. *P<0.05, **P<0.01; ns, not significant. *n*=3. (E) Colony growth of MCF10A^HER2^ and control cells in 0.3% ultra-pure agarose over 3 weeks. Five different-size colonies from ImageJ analysis were quantified. Representative microscopic images of colonies stained with Crystal Violet after 3 weeks are shown on the right. Statistical significance was calculated using unpaired two-tailed Student's *t*-test. *P<0.05, **P<0.01, ***P<0.001. Images are at 1.6× magnification. Scale bars: 1000 μm. *n*=3.

HER2 overexpression in non-tumourigenic MCF10A cells is a well-established breast cancer model and has been used in numerous *in vitro* studies (Muthuswamy et al., 2001; Imbalzano et al., 2009). To recapitulate the early transformational events and the stochastic nature of early breast cancer development, we generated a controllable *in vitro* model system by stably transducing a doxycycline-inducible HER2 construct in MCF10A cells (Carter et al., 2017). This model allows for the generation of transformed phenotypes in a synchronised and time-controlled manner and is useful for investigating early transformational events using multi-omic analysis (Fig. 1A). To analyse the range of HER2 expression at the protein level, we cultured cells for 24 h in five different concentrations of doxycycline, using ranges that have been used previously in inducible expression studies with other proteins (Baron et al., 1995; Leitner et al., 2014). In our model, a 24 h induction with 1 µg/ml doxycycline resulted in strong HER2 protein expression (Fig. 1B). When grown in three-dimensional (3D) cell cultures, control MCF10A cells (MCF10A^CTRL^) formed regular, spherical acini, whereas a majority of MCF10A^HER2^ acini were misshapen, with cells budding into the surrounding matrix (Fig. 1C; Fig. S1A). Our results indicate that activation of tyrosine kinases may promote the formation of these branched networks through the phosphorylation of activator phosphosites of FAK (Y576) and FAK (S574) via signalling of the MAPK pathway. We found CTTN to be highly and significantly phosphorylated at three sites – T401, S405 and T411 – and these sites are known to be activating post-translational modifications (Bandela et al., 2022). We propose that the molecular changes behind the disrupted acini are the result of abnormal HER2 expression that activates CTTN, which binds to FAK (PTK2), resulting in cell scattering by polymerisation of actin and loss of cell-to-cell communication (Dataset 1) (Kelley et al., 2011; Walkiewicz et al., 2015). HER2 overexpression resulted in significantly increased *in vitro* migratory and invasive potential, as measured by transwell assays (Fig. 1D) (Xiang and Muthuswamy, 2006; Paszek and Weaver, 2004). Furthermore, MCF10A^HER2^ cells displayed a hallmark of *in vitro* transformation, with increased anchorage-independent growth compared to that of control cells (Fig. 1E). Collectively, these results show that HER2 overexpression in MCF10A cells results in phenotypes associated with *in vitro* transformation. Indeed, aberrant expression of HER2 is already known to induce phenotypes associated with *in vitro* transformation (Seton-Rogers et al., 2004) and evokes aggressive tumorgenicity and metastasis *in vivo* (Alajati et al., 2013).

### Phosphoproteomic analysis following HER2 overexpression uncovers signalling changes associated with cancer

**Fig. 2.**
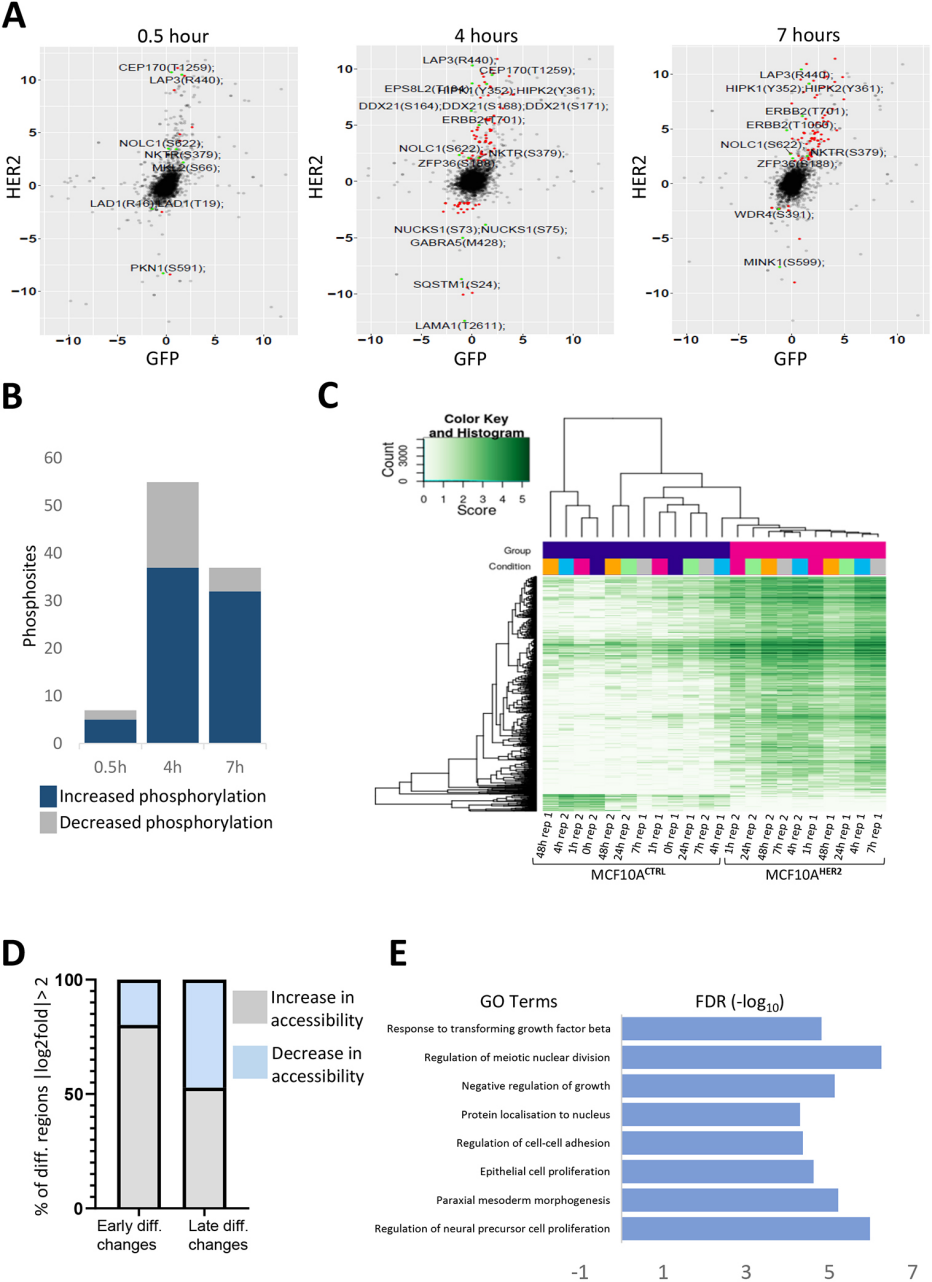
**HER2 promotes *in vitro* transformation through increase in signalling and widespread chromatin opening.** (A) Volcano plots depicting the phosphoproteome upon HER2 protein expression at 0.5, 4 and 7 h compared to control cells. Statistical significance is shown as log2 fold change for HER2>1.5, *P*<0.05, and log2 fold change for GFP<5, *P*>0.05. The plot shows the phosphosites that are significantly changing upon HER2 protein induction but not significantly changing in the GFP cells at the same time. Those with the highest increase or decrease in fold change are labelled. *n*=3. (B) Bar graph depicting the number of detected phosphosites increasing or decreasing in phosphorylation in the phosphoproteomic analysis at the indicated time points analysed. Statistical significance is shown as log2 fold change for HER2>1.5, *P*<0.05, and log2 fold change for GFP<5, *P*>0.05). (C) Differential accessibility (log2 fold change>0.5, FDR-corrected *P*<0.05) between MCF10A^HER2^ and control cells, plotted against the mean reads per region. Cells were grown in 3D cell culture from 0 to 48 h, and ATAC-seq was performed on their acini. Heatmap shows chromatin accessibility across all time points for each replicate in cells expressing HER2 or GFP (controls). *n*=3. (D) Fraction of total regions that are differentially accessible (up peaks) or inaccessible (down peaks) in early or late type comparisons. ‘Early’ time point represents data from 0, 1, 4 and 7 h combined. ‘Late’ time point represents data from 24 h and 48 h combined. Log2fold>2, FDR-corrected *P*<0.05. (E) Gene Ontology (GO) categories for biological processes for differential peaks that are significantly up (log2fold change>0.5, FDR-corrected *P*<0.05) for the early MCF10A^HER2^/early MCF10A^CTRL^ cells.

HER2 is a tyrosine kinase known to activate a plethora of signalling pathways downstream. To investigate the dynamic changes in the phosphoproteome over time, and the order in which they occur during the phased progression from normal to transformed cells upon HER2 overexpression, we performed an unbiased phosphoproteomic analysis of the early phosphorylation events (at 0.5, 4 and 7 h post HER2 protein induction). The experiment was carried out under standard growth conditions in two-dimensional (2D) cell culture, and without serum starving, to be closer to physiological conditions. A GFP-transduced MCF10A cell line was used as a control for doxycycline-only induced changes (MCF10A^GFP^). As expected, we observed an increase in HER2 phosphorylation levels in HER2 at T701 phosphosite and its family member EGFR (HER1) at Y1110 phosphosite (Fig. S1B). To filter changes relevant to HER2 induction, we selected only those phosphosites that were significantly changed upon HER2 expression but were not significantly changed in the MCF10A^GFP^ cells, with a stringent cut-off at log2 fold change for HER2>1.5, *P*<0.05, and log2 fold change for GFP<5, *P*>0.05 (Fig. 2A). From this refined dataset, some potential novel HER2 targets including NUCKS1 (S73 and S75), a frequently phosphorylated protein at multiple sites, were significantly downregulated at the 4 h time point (Fig. 2A), when HER2 protein levels were still quite low, as measured by western blotting (Fig. S1C). NUCKS1 is known to play a significant role in modulating chromatin conformation (Parplys et al., 2015; Grundt et al., 2004), and regulates events such as replication, transcription and chromatin condensation (Østvold et al., 2001). NUCKS1 phosphorylation at various phosphosites is also known to correlate with breast cancer resistance to retinoic acid, known to exert anti-proliferative effects in several breast cancer cell lines (Carrier et al., 2016). Other novel candidates include DDX21, with multiple phosphorylation serine sites (S164, S168 and S171), which were also significantly enriched in our phosphoproteomic analysis (Fig. 2A). We aimed to investigate the link between signalling and chromatin, and observed that DDX21-bound promoters on average had increased enrichment of active chromatin marks (H3K4me3, H3K27ac and H39Kac) but were depleted for repressive marks (H3K27me3 and H3K9me3) and promoter-distal (H3K4me1) marks (Calo et al., 2015). Some highly phosphorylated phosphosites, which have not been shown to be associated with HER2 protein expression, include homeodomain-interacting protein kinase 1 (HIPK1), which is highly expressed in invasive breast cancers (Park et al., 2012). SHC1 (S246), TTC7A (S182), CDC42EP3 (S89) and RIPOR1 (S351) were also significantly and stably activated at all the time points screened, suggesting that they may have important roles in the biology of HER2-expressing breast cancer cells (Fig. 2A; Fig. S2B). The effect of HER2 overexpression on all proteins was also quantified (Fig. 2B). Interestingly, of those changes, the 4 h time point showed the largest changes in phosphorylation, when HER2 levels were still quite low. Although HER2 protein expression was still low, some of these downstream changes might be present at later time points as part of the evolution process.

The low levels of HER2 activation at early time points may closely mimic, at least partially, the early signalling changes occurring in HER2-positive breast cancer patients. The signalling changes associated with low-level HER2 induction have not been evaluated to date. We re-analysed these data by decreasing the significance threshold to log2 fold change>0.5, false discovery rate (FDR)-corrected *P*<0.05 for HER2 expression, but not significantly changing for GFP (Dataset 1). This analysis revealed significant changes in phosphorylation in 1045 phosphopeptides over all time points in MCF10A^HER2^ cells, where the number of phosphosites increased in a time-dependent manner (Fig. S1D).

Using the DAVID Functional Annotation Tool (Huang da et al., 2009), and filtering for all significant changes (log2 fold change>0.5, FDR-corrected *P*<0.05) at all time points analysed, we identified the mitogen-activated protein kinase (MAPK) signalling pathway to be one of the most enriched cascades in our system (Fig. S1E). The idea that signalling has direct effects on chromatin has already been known, whereby receptor tyrosine kinases can relay extracellular signals by signal transduction pathways to the chromatin (Schreiber and Bernstein, 2002). Signalling pathways, particularly MAPK cascades, elicit modification of chromatin through various transcription factors and chromatin regulators (Clayton and Mahadevan, 2003; Pogna et al., 2010). Activation of the MAPK pathway ultimately leads to the phosphorylation of transcription factors, which is crucial for gene activation (Treisman, 1996). We hypothesised that the differentially regulated transcription factors and chromatin regulators identified in the phosphoproteomic screen are likely to contribute to chromatin changes mediating the transformed phenotypes. Indeed, our phosphoproteomic analysis revealed significant changes in various transcription factors known to affect chromatin dynamics (Fig. S1F). These chromatin regulators included SIRT1, SOX13, POU2F1, and multiple residues on POL2RA and NCOR1. In particular, the phosphorylation of JUN at residue S73 could be reconciled by a model in which phosphorylation of JUN triggers dissociation of histone deacetylases (HDACs) and facilitates the rearrangement of chromatin structure (Wolter et al., 2008). Based on these results, we then set out to assess, in an unbiased manner, the effects that signalling changes have on the chromatin organisation.

### Identification of two distinct chromatin accessibility landscapes within HER2-induced transformation

To investigate the interplay between signal transduction pathways and chromatin dynamics, we used an assay of transposase-accessible chromatin using sequencing (ATAC-seq) to determine the genome-wide chromatin accessibility landscape in the acini of MCF10A cells in a time-dependent manner (0-48 h) by isolating cells from 3D cell culture. Principal component analysis (PCA) separated the samples into two groups, ‘early’ (0, 1, 4 and 7 h time points) and ‘late’ (24 h and 48 h time points) (Fig. S1G). We selected these conditions with the aim of encompassing time points relevant to both types of analysis. The 0, 4 and 7 h time points were chosen to characterise early chromatin changes triggered by signalling. The late conditions were selected to detect the resulting delayed chromatin changes occurring later in the process of transformation. We identified 17,868 significant changes between MCF10A^HER2^ cells and control cells (T0 starting population before HER2 protein induction) over the time course, which showed an increase in accessibility in MCF10A^HER2^ cells relative to controls (Fig. 2C; Fig. S2A). We assessed differential accessibility between early and late groups and observed that a much larger fraction of regions, with >2-fold difference relative to T0, were enriched in the early group compared to in the late group (75% versus 44%, respectively; Fig. 2D). Conversely, only ∼2.9% of peaks in the early group and ∼6.5% of peaks in the late group were >4-fold more accessible, which we define as ‘hyper-accessible’ chromatin states (Fig. S2A). Even though the numbers of hyper-accessible versus hypo-accessible regions (which lose accessibility >4-fold) did not show a stark difference, the overall number of accessible regions following HER2 expression outnumbered inaccessible regions. This shows that there is an increase in chromatin accessibility during the early stages of transformation (Fig. 2D). Therefore, this might suggest that the first adaptive response to oncogenic HER2 signalling is altered chromatin accessibility to induce differential gene expression. Subsequently, the changes in chromatin accessibility even out in the later time points, with the number of hypo-accessible regions even exceeding the hyper-accessible ones at late time points, which could indicate that cells have reached an equilibrium (Fig. S2A).

**Fig. 3.**
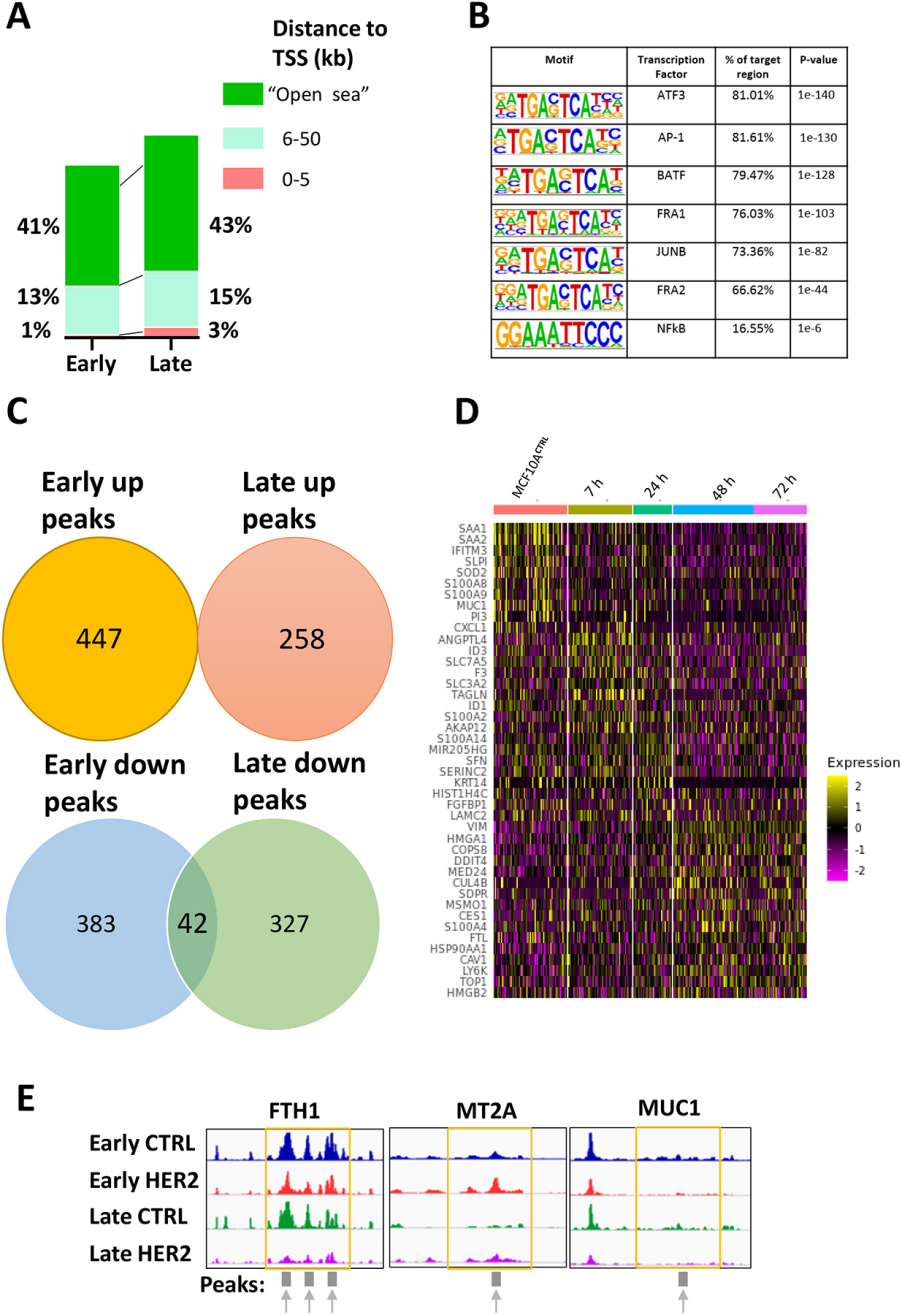
**Abnormal HER2 expression shows overlapping genes/transcription factors identified in multiomics data.** (A) Distance to closest transcriptional start sites (TSSs) of all differentially accessible regions in the early and late cell types. The bars represent only those regions that are upstream of the TSS. ‘Open sea’ refers to regions that are at least 50 kb or more upstream of the TSS. (B) Enrichment of transcription factor recognition sequences in differential ATAC-seq peaks comparing MCF10A^HER2^ and control cells based on HOMER analysis. Down peaks=log2fold<−2, FDR-corrected *P*<0.05. HOMER analysis using the accessible (up) peaks can be found in Fig. S2C. (C) Venn diagram showing the number of differentially accessible regions that are shared between the up (open) and down (closed) peaks in the early and late samples. Up peaks=log2fold>2, FDR-corrected *P*<0.05. Down peaks=log2fold<−2, FDR-corrected *P*<0.05. (D) scRNA-seq was performed in 2D cell culture on MCF10A cells with HER2 induction from 0 to 72 h (3 days). Heatmap summarises some of the most highly and lowly expressed genes with the induction of *HER2* gene. (E) Insertion tracks of samples at example regions. This signal is an average signal of three replicates of combined time points into either ‘early’ samples or ‘late’ samples. Differentially open regions are marked with arrows.

Next, we performed functional enrichment analyses [Gene Ontology (GO) terms] for upregulated peaks in the early HER2 signature (Fig. 2E). The regions with increased chromatin accessibility at all times analysed were enriched for GO terms associated with response to transforming growth factor, cell–cell adhesion, epithelial cell proliferation, morphogenesis and regulation of neural precursor cells. The differentially accessible regions upstream of the transcriptional start site (TSS) were largely gene distal, with relatively few promoter-proximal regions (Fig. 3A). To probe how the observed changes in cell signalling can underlie transcriptional and/or epigenetic control during cellular transformation, we examined transcription factor binding motifs that were significantly enriched in relation to all differential ATAC-seq peaks. The most significantly enriched motifs in the accessible chromatin regions as a result of perturbed HER2 expression were CEBP, HLF, ATF4 and CHOP (Fig. S2C). We also observed significant enrichment of motifs for all the time points analysed for inaccessible peaks corresponding to closed regions, which included ATF3, AP-1, BATF, FRA1, JUNB, FRA2 and NFκB (Fig. 3B). Previously, it has been shown that enrichment of AP-1 family member motifs is associated with increased accessibility (Hardy et al., 2016). There was some overlap between the family members of transcription factors identified in the phosphoproteomic screen and ATAC-seq motif analysis, including NFκB, JUN, ATF1, JUND and AP-1 (Fig. S2D). The transcription factors found in our motif analysis associated with accessible chromatin are known to be involved in several cancer types, including breast, lung, endometrial and prostate cancers with a more aggressive phenotype (Detry et al., 2008).

We next examined whether peaks were shared between those that were opening (more accessible) and those that were closing (less accessible) in the early and late groups. We found that there was a small overlap between early and late inaccessible peaks but none between the accessible peaks (Fig. 3C). This suggests that increasing accessibility is dynamic during transformation, and that sites with early loss in accessibility relative to T0 could potentially have driving roles in the population drift. We further examined the genomic distribution of the differentially inaccessible chromatin of the overlapping regions, which showed that most genomic regions were associated with two nearby genes (Fig. S2E). Namely, some of the common differential regions correlated with genomic location of FBN2, the genomic chromosomal coordinates of which were found to be matching with the promoter region of the *FBN2* gene. This gene was found to have aberrant promoter methylation in a number of cancers (Hibi et al., 2012) (Fig. S2E). Other regions included RIMS2, known to be associated with particularly aggressive breast cancers (Zhang et al., 2021), and APIP, which binds HER3 receptor, leading to the heterodimerisation between HER2 and HER3 and resulting in sustained activation of downstream signalling (Hong et al., 2016). No differentially accessible region was found to be promoter proximal, as all the regions were at least 5 kb upstream of the TSS (Fig. S2G).

To elucidate the heterogeneity in gene expression between subpopulations of cells in light of the pervasive chromatin opening we identified, we performed single-cell RNA sequencing (scRNA-seq) following induction of HER2 overexpression over 72 h. Cells were grouped according to their time point by Uniform Manifold Approximation and Projection (UMAP) dimensional reduction. Although there was no distinct separation between the time points, there was a trend in clustering of MCF10A^CTRL^- versus HER2-expressing cells (Fig. S3A). Seurat clustering found differentially expressed features and separated them into four groups, with cluster 0 enriching in the MCF10A^CTRL^ population, and cluster 1 associating with the highest HER2 expression (Fig. S3B). As expected, we observed a time-dependent increase in *HER2* gene expression (Fig. S3C). There is a consensus that high HER2 expression is associated with stem-like phenotype (Oliveras-Ferraros et al., 2010); however, much controversy remains on whether stemness and high-grade tumours are highly correlated with each other. Some studies have suggested a strong correlation between stemness and high oncogene expression, whereas others reveal little relationship (Poli et al., 2018; Šimečková et al., 2019). We identified clear transcriptional signatures of oncogenes associated with breast cancer progression such as the time-dependent increase in *ALDOA*, a gene that increases *in vitro* spheroid formation and increases abundance of cancer stem cells (Fig. 3D; Fig. S3D), and *LAMB3*, which mediates invasive and proliferative behaviours by the PI3K–AKT signalling pathway (Zhang et al., 2019), as well as the decrease in genes like *MUC1*, conversely, upon HER2 overexpression, the downregulation of which is linked to stem-like phenotype (Stingl, 2009). Although the expression of *ID3* is also associated with stemness (Huang et al., 2019), this pattern was not found in our data, suggesting that these processes overlap only partially.

Genome browser tracks of early and late HER2 samples showed the relative accessibility of some regions associated with the indicated gene, with arrow-marked regions in Fig. 3E indicating differentially open regions. The ferritin heavy chain (*FTH1*) gene, which displayed sharp decline upon HER2 expression (Fig. 3E; Fig. S3D) was also associated with inaccessible chromatin, as shown by the scRNA-seq and ATAC-seq datasets. Low *FTH1* expression is known to make breast cancer cells radiosensitive, and its higher expression is correlated with radioresistance (Tirinato et al., 2021). An in-depth analysis of *FTH1* expression in HER2-positive clinical samples might improve the efficacy of radiation treatments.

### Sustained low HER2 expression facilitates dedifferentiation and confers stem-like traits

**Fig. 4.**
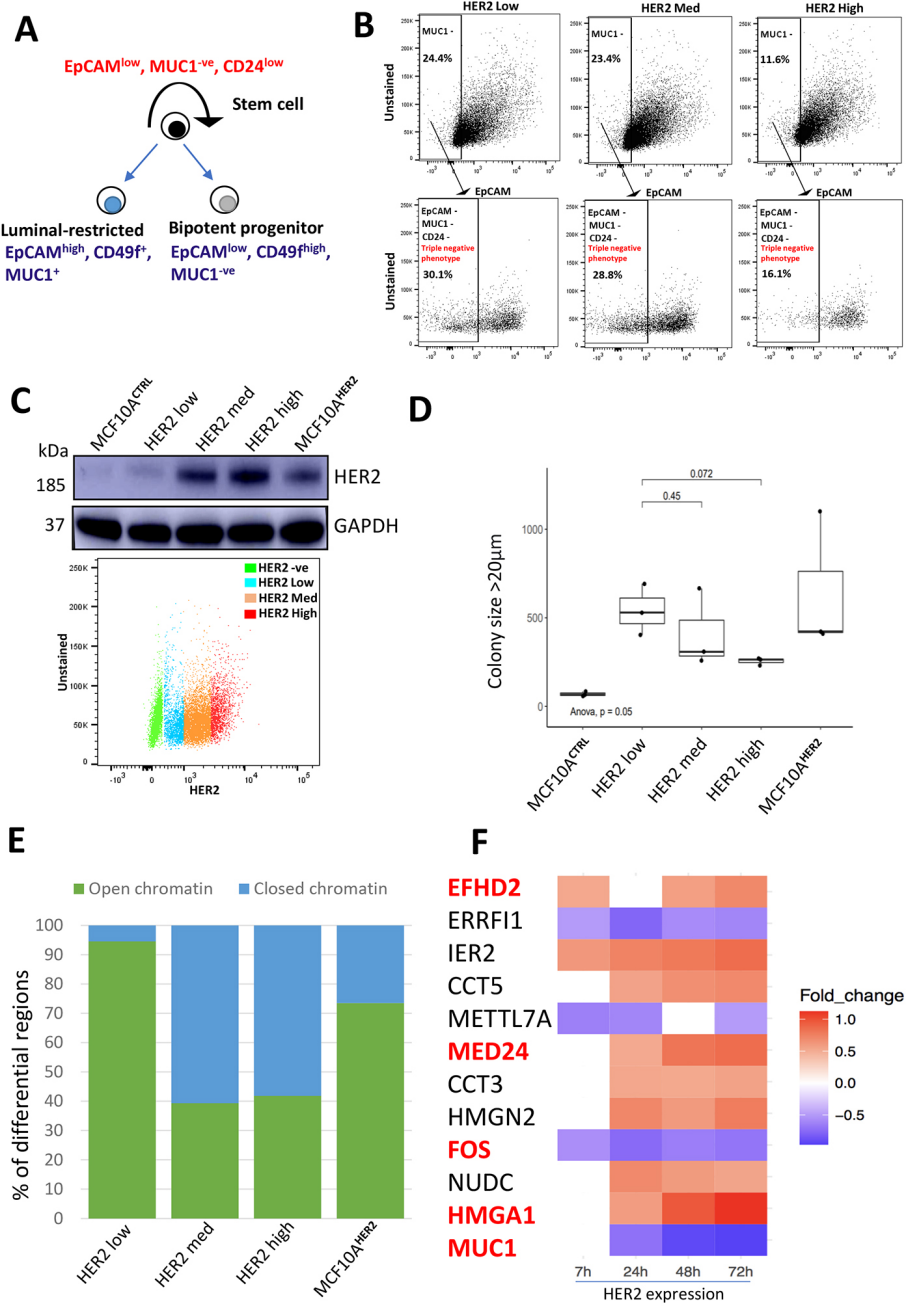
**Low HER2 expression leads to increased transformation, stemness and chromatin accessibility.** (A) Proposed simplified breast epithelial hierarchy present in human mammary glands. (B) Cells were analysed by flow cytometry, and HER2-positive cells were separated into three subpopulations of low, medium and high HER2 overexpression, as indicated. The enrichment of stem markers is shown as a proportion of the total number of cells exhibiting MUC1-negative and EPCAM-negative phenotype. The proportion of cells shown here shows the overlap between MUC1-negative and EPCAM-negative cells, all of which were subsequently 100% CD24 negative. *n*=3. (C) MCF10A^HER2^ cells were flow sorted into the labelled subtypes, and HER2 expression analysis in MCF10A cells by western blotting was performed. GAPDH was used as a loading control. The bottom 20% of HER2-expressing cells were labelled as ‘HER2 low’ cells (blue); the top 20% of HER2 expressing cells were labelled as ‘HER2 high’ cells (red). The middle population (35%) was labelled as ‘HER2 med’ (orange). HER2-negative cells are highlighted in green based on HER2-negative control cells. *N*=3. (D) HER2 expression was induced for 3 days, and cells were sorted based on HER2 expression into low, medium and high HER2 expression. 5000 cells from each condition were plated into ultra-pure agarose to investigate their *in vitro* transformative potential. Results are plotted as box plots from three biological replicates. Unpaired two-tailed Student’s *t*-test was performed to compare ‘HER2 med’ and ‘HER2 high’ groups to the ‘HER2 low’ group; *P*-values are displayed on the graph. One-way ANOVA was performed to determine statistical significance. The boxes represent interquartile range, and the whiskers indicate the minimum and maximum. *n*=3. (E) MCF10A^HER2^ cells were sorted into the three subtypes. ATAC-seq libraries were prepared and sequenced. DiffBind was used to analyse the differentially accessible regions, plotted as percentage of open or closed regions. *n*=3. (F) Heatmap shows genes of interest that are consistently differentially expressed in at least three of the four time points analysed upon HER2 overexpression. Blue rectangles represent genes that are downregulated; red rectangles represent genes that are upregulated. The white rectangles show lack of differential expression for that specific time point. Genes are only listed if the statistical significance had FDR-corrected *P*<0.05. Importance of genes highlighted in red is mentioned in the text.

MCF10A^HER2^ cells exhibited heterogeneous capacity for anchorage-independent growth when measured by their ability to form colonies in semi-solid medium, in that a significant proportion of MCF10A^HER2^ cells were able to form cell aggregates, with a >2-fold increase in colony-forming units compared to control cells (Fig. 1E). We hypothesised that cells possessing the ability to form colonies under anchorage-independent growth conditions are a selection of aggressive cells out of the total number of cells seeded. Conversely, the proliferative but non-malignant cells that often dominate any heterogeneous parental cell line would be selected against under these conditions. We evaluated whether anchorage-independent growth correlated with reprogramming-associated heterogeneity by testing the expression of proteins found in mammary epithelial stem cell hierarchy by flow cytometry (Stingl, 2009), in which it has been shown that breast stem cells are characterised by MUC1-negative, EPCAM^low^ and CD24^low^ expression (Fig. 4A). We therefore evaluated whether HER2 overexpression could enrich for cells with functional stem-like properties based on these three markers and found that this stem-like phenotype was enriched in MCF10A^HER2^ cells, as a large proportion of cells lost the expression of MUC1, EPCAM and CD24 (Fig. 4B; Fig. S3E and Fig. S4). Because our population is heterogeneous due to differing numbers of copies of the lentiviral HER2 construct, and we have the same amount of doxycycline used to induce the oncogene, the upper threshold of expression of HER2 will depend on the transgene copy number. We therefore hypothesised that stem-like markers would be positively correlated with HER2 levels in our heterogeneous population, i.e. cells having many HER2 copies would also be more likely to express stem-like markers. Surprisingly, we found that cells expressing relatively low HER2 levels had the most pronounced stem-like phenotype compared to other flow-sorted populations of cells with increasing levels of HER2 (Fig. 4B). We confirmed the different levels of HER2 protein expression after sorting cells into three compartments of low, medium and high HER2 expression by western blotting, which correlated as expected (Fig. 4C). We also compared these subpopulations of cells to HER2-positive patient samples that were already known to be HER2 positive with immunohistochemical scores of 3+ or 2+, which showed that low HER2-expressing cells expressed even less HER2 protein compared to these groups (Fig. S3F). Next, to determine the transformational potential of these cell types by measuring anchorage-independent growth, we flow sorted MCF10A^HER2^ cells into the three different cell populations and paradoxically found that low HER2-expressing cells had increased transformational potential relative to that of the other populations of sorted cells (Fig. 4D). We thought that high HER2-expressing cells may be undergoing oncogene-induced senescence (OIS), thus resulting in reduced colony formation compared to other cell types. To confirm this, we measured proteins implicated in OIS but found no significant increase in OIS markers in the high HER2-expressing cells compared to other populations, indicating other biological effects being responsible for the lower capacity in anchorage-independent growth of high HER2-expressing cells (Fig. S3G). It is possible that high oncogene expression induces cancer cells to dormancy that is associated with loss of ability to self-replicate and differentiate (Bellovin et al., 2013).

Because we found that chromatin opening was the feature associated with early signalling to chromatin response, we wanted to know whether this was reflected in the phenotypic heterogeneity, in particular low versus high HER2 levels. To this end, we used ATAC-seq to determine the genome-wide chromatin accessibility landscape in the five different populations of cells (MCF10A^CTRL^, low HER2, medium HER2, high HER2 and MCF10A^HER2^ cells). We analysed these data by comparing each cell type to the control cells (MCF10A^CTRL^) and comparing the percentage of differentially accessible regions between the cell types. We found that low HER2-expressing cells exhibited the highest percentage of chromatin opening compared to other cell populations (Fig. 4E), confirming that the phenotypes associated with invasiveness and anchorage-independent growth were driven by molecular features in stem-like cells and opening of chromatin. To put the magnitude of these chromatin differences in context, i.e. the differential accessibility between low HER2- and high HER2-expressing cells, we found that ∼95% of peaks were accessible in low HER2-expressing cells. Conversely, only ∼42% of the peaks were open (accessible) in the high HER2-expressing cells. Overall, these data indicate that a sharp increase in HER2 may result in triggering cell intrinsic defensive systems, whereas a low-level sustained presence of HER2 can shift cell identity, via chromatin remodelling, towards tumour-promoting phenotypes.

We found that a subset of these scRNA-seq-unique differentially expressed genes that were either upregulated or downregulated at multiple time points were also associated with heterogeneity of breast cancer, related to cancer progression and stem cells (Fig. 4F). For example, expression of *HMGA1*, which is known to promote breast cancer angiogenesis through the transcriptional activity of *FOXM1* (Zanin et al., 2019), increased in a time-dependent manner (Fig. 4F). However, expression of *FOS*, a pro-proliferative transcription factor, which has been validated in breast tumour samples and is highly expressed in relapse samples and treatment failures (Vendrell et al., 2008), was found to be downregulated at all time points (Fig. 4F). Intriguingly, high proliferation rates as a result of *FOS* expression can lead to improved outcomes for patients with breast cancer, as they can lead to higher expression of apoptosis-effector genes (Fisler et al., 2018). Our data also show the time-dependent increase in *EFHD2*, a gene linked to epithelial–mesenchymal transition and metastasis (Fan et al., 2017). *MED24*, encoding a subunit for the mediator complex of RNA polymerase II, is known to be a downstream target of HER2 and may be a critical gene required for cancer development (Liu et al., 2019).

## DISCUSSION

In this study, we addressed the question of what the earliest molecular changes are at the interface between increasing oncogenic HER2 signalling and chromatin accessibility in a non-transformed breast epithelial cell line. Overexpression of the *HER2* oncogene in breast epithelial cells resulted in some unexpected changes in cellular phenotypes. Namely, we observed an inverse relationship between HER2 levels and tumourigenic properties *in vitro*, where cells expressing a sub-threshold amount of HER2 protein exhibited increased anchorage-independent growth. This was also associated with features of dedifferentiation towards breast stem cell identity. Among the expected features, MCF10A^HER2^ cells underwent *in vitro* transformation, as evidenced by increased anchorage-independent growth accompanied by the formation of spindle-like conformations in 3D cell culture (Fig. 1B,D). These findings are concordant with other studies in which loss of cell polarity following HER2 overexpression has been described (Ortega-Cava et al., 2011; Hartman et al., 2013; Xiang and Muthuswamy, 2006).

We propose that a sub-threshold level of HER2 protein has the ability to elicit activation of signalling pathways that directly impact on chromatin to drive dedifferentiation and survival and to enhance transformation. Although high levels of oncogenic expression are an important biomarker in diagnosing HER2-positive breast cancer, our data support the hypothesis that even low levels of HER2 protein expression can be associated with disease aggressiveness, poor patient outcome and therapeutic resistance (Gilcrease et al., 2009). The mechanism underlying why low HER2-expressing cells can be aggressive and its prognostic value have not been sufficiently evaluated. Our data show that the subset of low HER2-expressing cells likely use changes in chromatin state as their route for cellular transformation (Fig. 4E); the accessible chromatin induced by low-level HER2 signalling may continuously predispose cells to secondary additional hits required for metastasis and therapeutic resistance (Denny et al., 2016). The resulting chromatin changes via low HER2 expression may create a lasting and highly transformative state. It should be noted that early HER2 protein levels, as measured by the timing of HER2 expression from basal levels, is not the same as low HER2 protein levels, as the latter does not activate signalling pathways.

Across the different subtypes of breast cancers, and in particular HER2-positive breast cancer, loss of differentiation is associated with lower patient survival and aggressiveness (Margaryan et al., 2017; Pupa et al., 2021). However, in low HER2-expressing cells, the correlation between dedifferentiation and aggressiveness remains unclear. Stem marker signatures drive cancer growth, and their inhibition delays it (Rudin et al., 2012). Several known stem markers, including the EPCAM, MUC1 and CD44 signatures, promote transformation and tumour progression (Stingl, 2009). Our data suggest that changes in chromatin and dedifferentiation correlate with the aggressive nature of the low HER2-expressing cells, but the mechanism underlying this process has yet to be identified (Fig. 4B).

We observed leucine aminopeptidase 3 (LAP3) to be significantly activated in our phosphoproteomic screen at all of the time points analysed in MCF10A^HER2^ compared to MCF10A^GFP^ cells (Fig. 2A). LAP3 is known to play a critical role in breast cancer cells by regulating migration and invasion, and is associated with metastasis (Fang et al., 2019). In addition, we found that phosphorylation of nucleolar and coiled-body phosphoprotein 1 (NOLC1) at residue S622 was also significantly increased at all time points (Fig. 2A). This protein is highly expressed in nasopharyngeal carcinomas (Hwang et al., 2009) and in breast cancer cells (Sacco et al., 2016). The consistent and highly stable activation of these two proteins may serve as potential biomarkers for late-stage disease and provide important targets for antimetastatic therapeutic targets. Furthermore, zinc finger protein 36 (ZFP36) is correlated with lower-tumour grade breast cancer (Canzoneri et al., 2020). Interestingly, we found that ZFP36 (S188) was significantly activated at the 4 h and 7 h time points but not at the earlier 30 min time point (Fig. 2A), indicating that low HER2-expressing cells prefer a programme of signalling phosphosites associated with worse patient outcome (Canzoneri et al., 2020).

The morphological changes in breast cancer models are often used to indicate the high transformational characteristics of those cells (Barker et al., 2022). We found that proteins associated with aggressive basal-like phenotype were found to be increased in our phosphoproteomic screen, which included ADGRA2 (S1079) and DENND4C (S1250). This shows that the morphological changes observed in our system (Fig. 1B) were likely to be caused by HER2-induced transformation.

It is possible that the intrinsic heterogeneity found within the tumour population may be preventing specific patterns from emerging in a bulk RNA-sequencing analysis. It is known that differential downregulation of IFITM family members is associated with resistance maintenance following anti-HER2 therapy, trastuzumab (Wang et al., 2019). Our scRNA-seq data reveal downregulation of IFITM3 within 24 h of HER2 overexpression, that is maintained until at least 72 h, which could show that this does not decrease as a result of resistance but may predispose resistance to therapies at the very early stage of disease. Overall, our data show the power of combining genome-wide molecular approaches using an *in vitro* transformation model system to uncover subtle but relevant variations in cellular states. Given the dramatic remodelling of the chromatin state driven by a single factor in HER2-positive breast cancer, we speculate that other cancer types may also feature similar mechanisms of cellular transformation through chromatin remodelling. Cataloguing early chromatin changes can emerge as a promising therapeutic target, with a particular focus on early and low HER2-induced alterations in breast cancer. We attempted to integrate the ATAC-seq data with the signalling changes we have observed to see whether there is a strict linear correlation; however, within the confines of the small number of time-point pairs available for analysis, it is not appropriate to correlate the ATAC-seq and phosphoproteomic timeseries data to try to identify biological mechanisms, as the problem of multiple testing would make such correlations almost meaningless.

Metastasis is a multi-step, low-probability process, in which primary cells must invade the local tissue and extravasate into a distant site. Our work shows that low HER2-expressing cells gain transformational ability through dedifferentiation and dramatic chromatin remodelling. This model could be further extended to assess how HER2-driven changes in chromatin state are used as a route for metastasis in *in vivo* models, and whether low loss of differentiation correlates with aggressiveness in more physiologically relevant models.

## MATERIALS AND METHODS

### Cell culture

The immortalised human mammary epithelial cell line MCF10A was obtained from the American Type Culture Collection (ATCC) and grown under recommended conditions. Briefly, MCF10A cell medium consists of Dulbecco's modified Eagle medium (DMEM/F12) (Sigma-Aldrich, #D8347) supplemented with 5% horse serum (Sigma-Aldrich, #H1138), 0.5 µg/ml hydrocortisone (Sigma-Aldrich, #H0888), 20 ng/ml epidermal growth factor (EGF) (Sigma-Aldrich, #E4127), 100 ng/ml cholera toxin (Sigma-Aldrich, #C8052), 10 µg/ml insulin (Sigma-Aldrich, #i9278) and 1× penicillin/streptomycin (Pen/Strep).

HEK293T cells were cultured in DMEM (Sigma-Aldrich, #D5796) in 10% foetal bovine serum (FBS) with 1× Pen/Strep.

For 3D overlay cell cultures, cells were grown in chamber wells in a mixture of Matrigel (Corning, #356230) and collagen (Corning, #11563550), which were mixed with 0.1* *M NaOH and 10× PBS, as previously described (Xiang and Muthuswamy, 2006). To collect cells from 3D cell cultures, cell recovery solution (Corning, #354253) was used at 4°C for 30-60 min according to the manufacturer's instructions. Staining 3D acini were fixed with 4% paraformaldehyde (PFA). Acini were permeabilised with 0.5% Triton X-100 and blocked in 10% goat serum in PBS-Tween 20. Acini were stained with phalloidin dye overnight at 4°C. The detachable chambers were removed, and acini were mounted in mounting media reagent and allowed to dry in the dark at room temperature. Once dried, slides were visualised using a fluorescence microscope.

### Vectors and viral infections

To generate the HER2-inducible MCF10A cell line (Carter et al., 2017), we first transiently transfected HEK293T cells using jetPRIME transfection reagent (Ppolyplus, #114-15). The inducible HER2 construct (Addgene, #46948) alongside pMD2.G (Addgene, #12259) (envelope plasmid), and of pCMV delta R8.2 (Addgene, #12263) (packaging plasmid) were transfected into 90% confluent HEK293T cells for 24 h. Lentiviral particles were harvested by centrifugation, and early-passage MCF10A cells were infected for 48 h. Cells were then flow sorted based on GFP expression to obtain a pure population.

### Western blotting

Cells were harvested and lysed in RIPA buffer containing protease and phosphatase inhibitors. Lysates were mixed with sample loading buffer, and proteins were resolved using sodium dodecyl sulphate-polyacrylamide gel electrophoresis (SDS-PAGE) and transferred onto PVDF membranes. Membranes were blocked in 5% milk, and antibodies were incubated overnight in 5% bovine serum albumin (BSA) solution. Antibodies used included anti-HER2 (Cell Signaling Technology, #2165, 1:1000), anti-GAPDH (Cell Signaling Technology, #2118, 1:2500), anti-p53 (Cell Signaling Technology, #2527, 1:1000), anti-p27 (Cell Signaling Technology, #3836, 1:1000), anti-p21 (Cell Signaling Technology, #2947, 1:1000), anti-tubulin (Abcam, #7291, 1:1000) and anti-rabbit secondary (Amersham ECL Rabbit IgG, HRP-linked whole Ab, #NA934, 1:5000).

Human samples were obtained from Barts Cancer Institute tissue bank. Where human samples were used, informed consent was obtained from all individual participants included in the study. All clinical investigations were conducted according to the principles expressed in the Declaration of Helsinki.

### Flow cytometry and flow sorting

Cultured cells were detached from plates with trypsin and stained with 2% horse serum. Cells were then stained with the following conjugated antibodies: anti-HER2 BV650 (BD Biosciences, #745299, 1:100), anti-EPCAM APC (BD Biosciences, #347200, 1:40), anti-MUC1 BV786 (BD Biosciences, #743410, 1:50), anti-CD24 BV711 (BioLegend, #311135, 1:50) for 20 min at room temperature. Cells were washed with 1 ml of 2% horse serum and then resuspended in 4′,6-diamidino-2-phenylindole (DAPI) solution. Stained cells were analysed on a BD LSRFortessa™ cell analyzer (BD Biosciences). For cell sorting, cells were stained with the antibodies of interest and isolated using an ARIA fusion cell sorter.

### ATAC-seq library preparation and differential analysis

Cells (5×10^5^) were directly recovered from cell culture by trypsin from 2D cell culture or by using the recovery solution (Corning, #354253) for cells grown in either 2D or 3D cell culture. ATAC-seq libraries were generated as described previously (Buenrostro et al., 2015), with minor amendments. We performed ten initial PCR amplification cycles followed by direct purification of the transposed DNA, without performing quantitative PCR to calculate the additional numbers of required cycles. Sequencing data were aligned to the human genome (grch38) using bowtie2. Peaks were called on each biological replicate of all ATAC-seq reads using MACS2, and putative copy number and mitochondrial regions were removed. The peak dataset for differential analysis was generated by applying a threshold using a desired fold-change and a –log10-transformed FDR-adjusted *P*-value. Differential accessibility was assessed using DiffBind (https://bioconductor.org/packages/devel/bioc/vignettes/DiffBind/inst/doc/DiffBind.pdf), and regions were called differentially accessible based on log2 fold change and FDR-corrected *P*-value.

### Phosphoproteomic sample preparation

For phosphoproteomic experiments, cells were grown in 2D cell cultures. Cell pellets were lysed using 8* *M urea lysis buffer (containing phosphatase inhibitors). The amount of protein in the lysates was quantified by bicinchoninic acid (BCA) assay. Then, 250 µg from each sample was digested into peptides with immobilised TPCK-trypsin beads (Thermo Fisher Scientific, #20230) at 37°C overnight. Phosphorylated peptides were enriched from total peptides using TiO_2_ chromatography, as reported previously (Montoya et al., 2011; Larsen et al., 2005). Finally, peptides were snap frozen and dried in a SpeedVac. Dried peptides were dissolved in 0.1% trifluoracetic acid and analysed by liquid chromatography– tandem mass spectrometry (LC-MS/MS) on a Q Exactive plus mass spectrometer (Thermo Fisher Scientific). Peptide identification was performed using the Mascot search engine (Casado and Cutillas, 2011). Allowed variable modifications were phosphorylation on Ser, Thr and Tyr, and oxidation of Met, and Pescal software (Casado and Cutillas, 2011; Cutillas and Vanhaesebroeck, 2007) was used to quantify the peptides. Kinase-substrate enrichment analysis (KSEA) (Casado et al., 2013) was used to determine kinase activities. The intensity values were calculated by determining the peak of each individual extracted ion chromatogram and plotted as heatmaps. The resulting quantitative data were transferred and visualised in Microsoft Excel. The significance (log2 fold change<−0.5-fold, FDR-corrected *P*<0.05 for downregulated phosphosites and log2 fold change>0.5-fold, FDR-corrected *P*<0.05 for upregulated phosphosites) of each phosphosite was annotated by an asterisk; we used the ‘filter’ function in Excel to filter out those phosphosites that were not significant. All of the significant MCF10A^GFP^ data were filtered out, while simultaneously filtering out non-significant data for the MCF10A^HER2^ cells, giving us significant changes in MCF10A^HER2^ cells that were not significantly changing in the MCF10A^GFP^ cells. The number of phosphosites was determined by the number of columns as each column contained one phosphosite, unless overlapping sites were present, in which case they were manually counted.

### Migration/invasion assays

A chilled Matrigel or collagen mixture was directly pipetted on the centre of 8 μm pore size transwell inserts (Millicell, #MCEP12H48) that were placed into a 12-well plate, and allowed to solidify at 37°C. Meanwhile, cells were trypsinised and pipetted onto the transwell inserts – which were either coated with matrix or left uncoated – and cultured for 16 h. Highly migratory/invasive cells were stained with 0.05% Crystal Violet dye. Images of random regions were taken using a standard light microscope and quantified using ImageJ.

### Soft agar colony formation assays

A 0.8% base layer was formed in plates using ultra-pure culture grade agarose (Thermo Fisher Scientific, #16500500) allowed to settle at room temperature. Five-thousand cells per well were mixed with 0.3% agarose and plated evenly, drop wise, on top of the base layer. Medium was changed every 2 days for 3 weeks. Colonies were fixed using 4% PFA and permeabilised using 100% methanol. Colonies were stained using 0.05% Crystal Violet dye, and images were taken using a dissecting microscope. Binary masks were applied to each of the images, and thresholding parameters for diameter ranging from 10 μm to 100 μm were set on ImageJ. Colonies were counted using ImageJ only if they satisfied criteria above the threshold values, and colony counts were then manually checked and adjusted if necessary.

### scRNA-seq

MCF10A cells were induced with 1 μg/ml doxycycline at 0, 7, 24, 48 and 72 h in 2D cell cultures. Cells were then detached using TrypLE (Gibco) and collected in 1× Dulbecco's PBS (DPBS; Gibco). After one wash in 1× DPBS, cells were resuspended in 2% BSA-DPBS at a concentration of 10,000 cells/μl. Then, 500,000 cells (50 μl) were blocked with 10 μl TruStain FcX blocking solution (BioLegend). Each treatment group was stained with 0.5 μl specific TotalSeq™-A Hashtag antibodies and 0.5 μl TotalSeq™-A0133 anti-human CD340 (ERBB2/HER2) protein expression antibody. Cells were washed three times with 1 ml of 2% BSA-DPBS and resuspended to a concentration of ∼10,000 cells/µl. Equal volumes of each treatment group were pooled, and cell pool was assessed for cell concentration and viability. Single-cell cDNA, protein expression (ADT) and hashtag (HTO) libraries were generated using Chromium Single Cell 3′ version 2 reagents (10× Genomics and BioLegend) as per the manufacturers' protocols. Single-end sequencing of libraries was performed by Novogene on a Novaseq 6000 (Illumina) sequencer with HTO libraries constituting 5% of the sample.

Single-cell data were run through the 10× Genomics CellRanger pipeline to produce count tables for gene expression, HTO counts for sample identification and ADT counts for HER2 expression. Cells were identified and assigned to a time point using the HTO counts table and the HTODemux method in Seurat. To exclude cells that did not respond to the doxycycline induction, treated cells with less than 35 counts of the ERBB2/HER2 expression tag were filtered out. The remaining gene expression data were run through Seurat's basic data processing pipeline. The data were normalised and scaled, and the effects of the cell cycle were regressed out using Seurat's cell cycle regression strategy. The data were then run through PCA. The principal components were used to identify clusters, and UMAP was run for visualisation. Two different differential expression analyses were run using Seurat's FindAllMarkers function, one across the different clusters and one across the different time points.

### ATAC-seq bioinformatics analysis pipeline

The ATAC-seq data were provided as FASTQ files. Quality control of raw sequencing read files was performed using FastQC. Illumina adapter trimming was done using Cutadapt with the following settings: Cutadapt -a CTGTCTCTTATACACATCT -A CTGTCTCTTATACACATCT -o out.1.fastq -p out.2.fastq. Trimmed reads were aligned using the human genome, Genome Reference Consortium Human Build 38 patch release 13 (GRCh38.p13), using bowtie2, and a SAM file was obtained with the following settings: bowtie2 index −1 trimmed FASTQ file −2 trimmed FASTQ file –S 1.sam. The resulting SAM files were converted into binary bam files (setting: Samtools view –Sb in.samfile>out.bamfile), sorted (setting: Samtools sort in.bamfile -o out.bamfile) and indexed (setting: Samtools index in.bamfile). To ensure an improved mapping quality, we removed mitochondrial DNA with the following settings: Samtools view –h in.bamfile | removeChrom - - chrM | Samtools view - b ->out.bamfile. PCR duplicates were removed from the files using Picard tools with the following settings: Java -jar picard.jar MarkDuplicates I=in.bamfile O= out.bamfile M=dups.txt REMOVE_DUPLICATES=true VALIDATION_STRIGENCY=LENIENT.

For viewing samples on genome bowser or assessing reproducibility and data exploration, all samples were ‘downsampled’ to the same number of reads with the following settings: samtools view -b -s [downsampling_ratio] in.bam>out.downsampled.bam. Peaks calling was done for each individual non-downsampled file with MACS2 ‘callpeak’ with the following settings: MACS2 callpeak -t inbamfile -f BAMPE -n in.bamfile -g ce –keep-dup all. These files were then analysed using DiffBind for differential analysis on R. For each sample, a path to the peaks and the bam file were listed in Microsoft Excel and loaded in R [setting: db.object=dba(sampleSheet=“name_of_sample_sheet”)]. Then, the next step was to take the alignment files and compute count information for each of the peaks/regions in the consensus set with the settings db.object=dba.contrast(db.object, categories=DBA_TREATMENT, block=DBA_CONDITION, minMembers=2) and db.object=dba.analyze(db.object,bParallel=TRUE,method=DBA_ALL_METHODS). R was used to plot the differential changes such as MA plot with an appropriate threshold [setting: dba.plotMA(db.object,th=“0.05”,method=DBA_DESEQ2)]. Significant changes could then be saved from up or down peaks, e.g. with the setting up_peaks_db.object.SigChanges.0.05FDR <- db.object.SigChanges.“0.05FDR”[db.object.SigChanges.0.05FDR$Fold>0,], and counted using the command line, and were plotted as percentages in Prism or Microsoft Excel in the form of a chart/graph. Motif enrichment analysis was performed using Hypergeometric Optimization of Motif EnRichment (HOMER) (Heinz et al., 2010).

## Supplementary Material

10.1242/dmm.049894_sup1 Supplementary information
