# Supplementary material for: Low HER2 expression in normal breast epithelium enables dedifferentiation and malignant transformation via chromatin opening
Source: Dis Model Mech. 2023 Feb 1;16(2):dmm049894. doi: 10.1242/dmm.049894 (PMC9922733; doi:10.1242/dmm.049894)
Supplement: Supplementary information [file dmm-16-049894-s1.pdf]

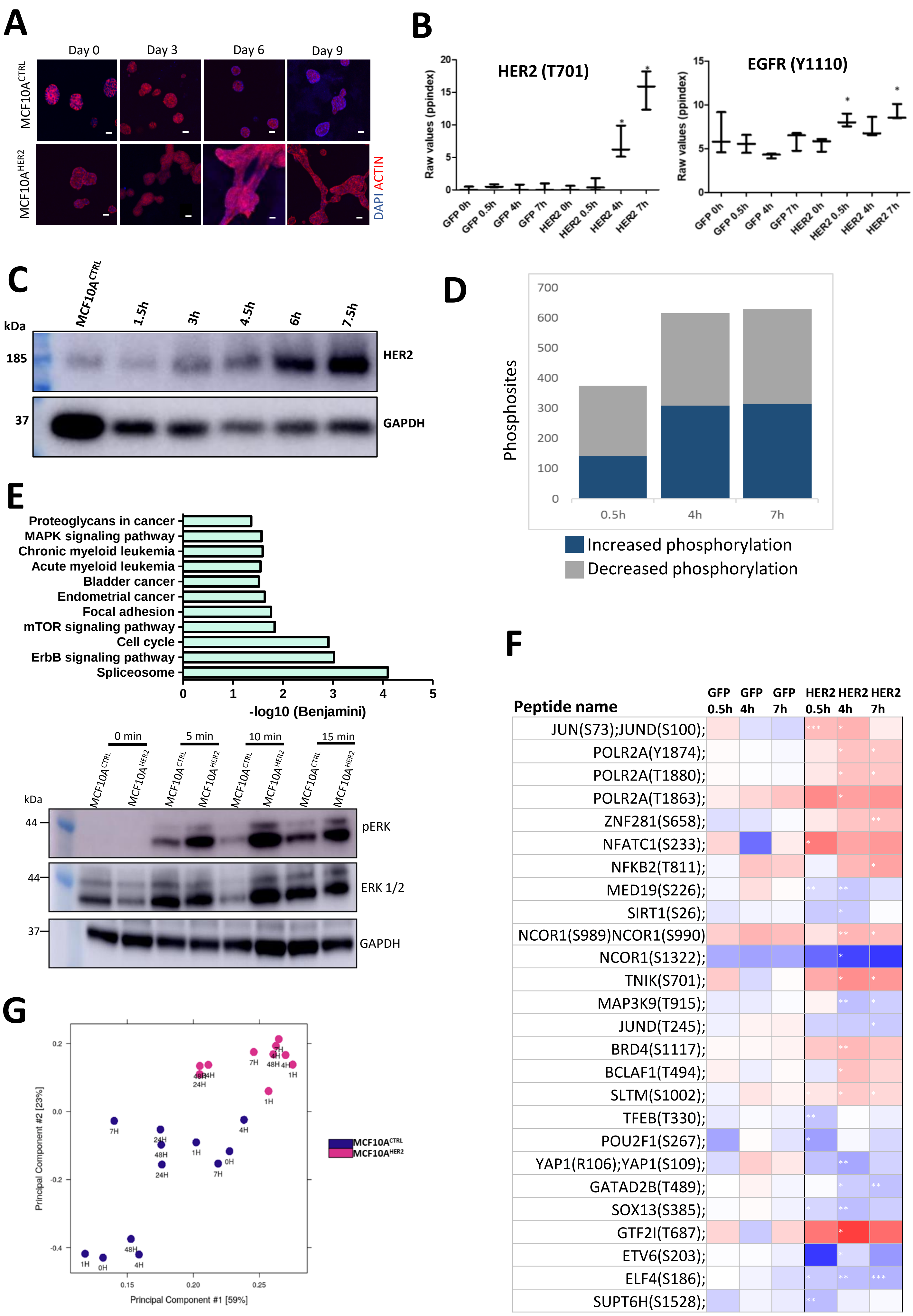

Disease Models & Mechanisms • Supplementary information

Fig. S1. HER2 expression is necessary and sufficient for transformational ability and signalling *in vitro*.

(A) MCF10AHER2 and control cells were cultured in 3D cell culture over 9 days. Control cells formed spherical acini which increased in size over time. MCF10AHER2 cells formed flat projecting cells of complex masses, typical of transformed cells. Images captured by confocal, LSM 510 microscope. Scare bars represent 100µm. N=3.

(B) An internal quality control (QC) for phosphoproteomic analysis. HER2 phosphorylation modification (T701) increases in a time dependent manner. EGFR [HER1] (Y1110), a family member of HER2, also becomes marginally activated in a time dependent manner compared to control cells. [* FDR corrected p-value of < 0.05].

(C) Western blot analysis of HER2 protein in a time-dependent manner in the early time-points upon induction with the same concentration of doxycycline (1µg/ml) from 0h to 7.5h.

(D) Bar graph depicting the number of detected phosphosites increasing or decreasing in phosphorylation in the phosphoproteomic analysis at the time-points analysed. Significance is shown to log2fold change > 0.5, FDR corrected p-value of < 0.05. This graph shows analysis performed using lower statistical threshold compared to figure 2B.

(E) Signalling pathway analysis of the early immediate changes in transformation. Signalling pathway analysis using the DAVID KEGG PATHWAY tool of the differentially phosphorylated events at all time points investigated upon HER2 protein induction is shown. To detect ERK activation MCF10ACTRL and MCF10AHER2 cells were gown in serum starved cell media for 24 hours and then stimulated with full media for the indicated time points or left in the serum starved media as a negative control. Detection of phospho-ERK (Thr202/Tyr204) is shown. After stripping, the same membrane was blotted for total-ERK (ERK 1/2) and GAPDH was used a loading control. N=2.

(F) Identification of transcription factors and chromatin regulators. A list of transcription factors and chromatin regulators becoming differentially phosphorylated upon HER2 expression in at least one time-point that are not significantly changing in GFP-transduced MCF10A cells. [* FDR corrected p-value of < 0.05, **FDR corrected p-value of < 0.001, *** FDR corrected p-value of < 0.001].

(G) Principal component analysis (PCA) of all samples used in this study. Samples are colour-coded by cell type.

Disease Models & Mechanisms • Supplementary information

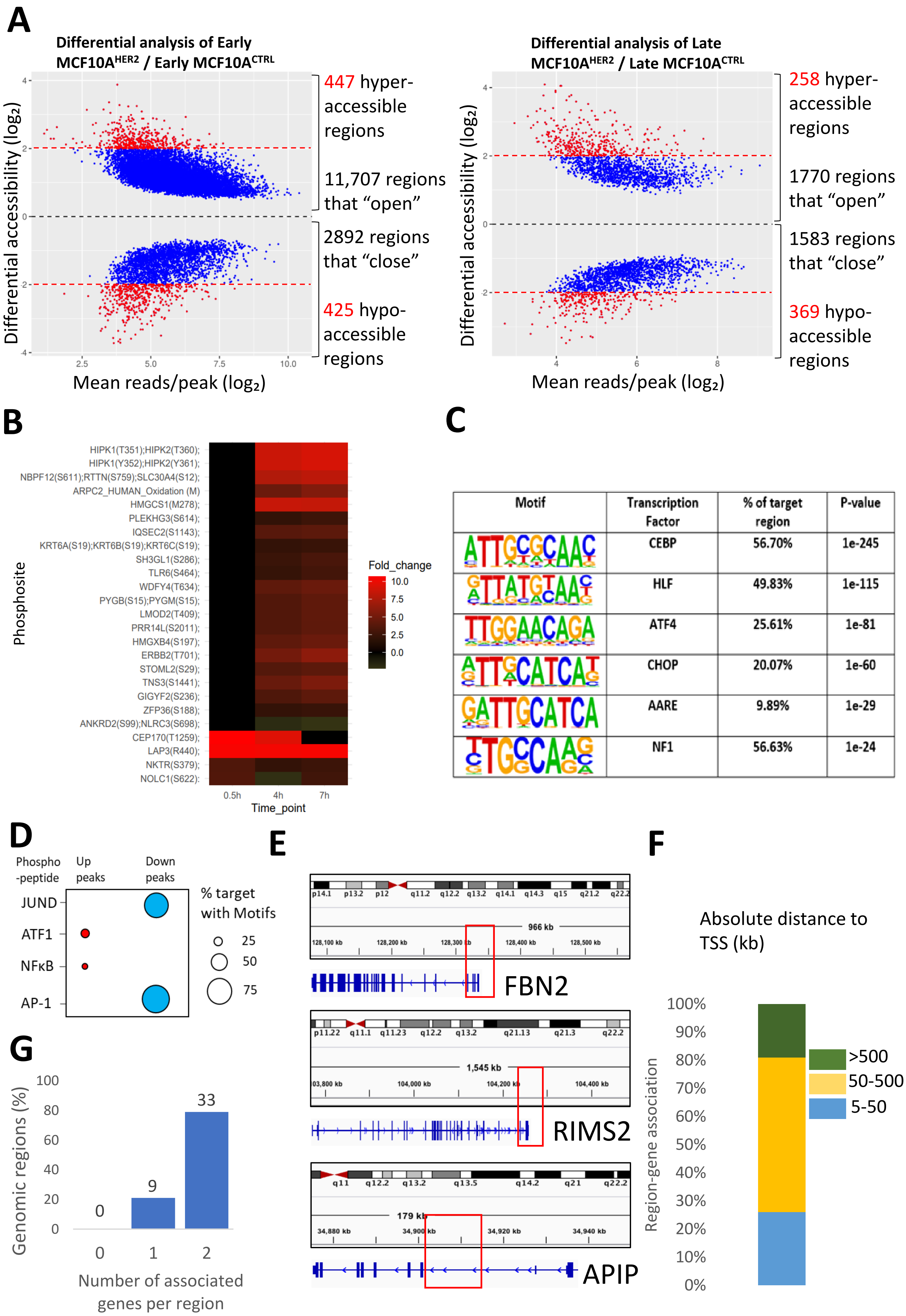

Motif	Transcription Factor	% of target region	P-value
	CEBP	56.70%	1e-245
	HLF	49.83%	1e-115
	ATF4	25.61%	1e-81
	CHOP	20.07%	1e-60
	AARE	9.89%	1e-29
	NF1	56.63%	1e-24

Disease Models & Mechanisms • Supplementary information

Fig. S2. Early HER2 expression drives a dramatic increase in chromatin accessibility compared to normal cells.

(A) Differential accessibility (log2 fold change in reads per accessible region) between MCF10A$^{\mathbf{HER2}}$ and control cells, plotted against the mean reads per region. Cells were grown in 3D cell culture from 0-48 hours and ATAC-seq performed on their acini. “Early” time-points represents 0h, 1h, 4h, and 7h data combined. “Late” time point represents 24h and 48h time-points combined. Each dot represents a region, with the blue dots representing a log2fold change of at least 0.5.

(B) Proteins that become phosphorylated consistently in at least 2 out of the 3 different time-points analysed.

(C) Enrichment of transcription factor recognition sequences in differential ATAC-seq peaks comparing MCF10A$^{\mathbf{HER2}}$ and control cells based on HOMER analysis using the accessible (up) peaks.

(D) GREAT database analysis showing the number/percentage of genes associated per region of the common regions found between the early up and late up peaks in the ATAC-seq data .

(E) Absolute distance to closest transcription start sites (TSSs) of the common differentially accessible regions in the early up and late up peaks.

(F) Single cell expression of MCF10A cells in UMAP. Cells are colour coded according to the time-points indicated.

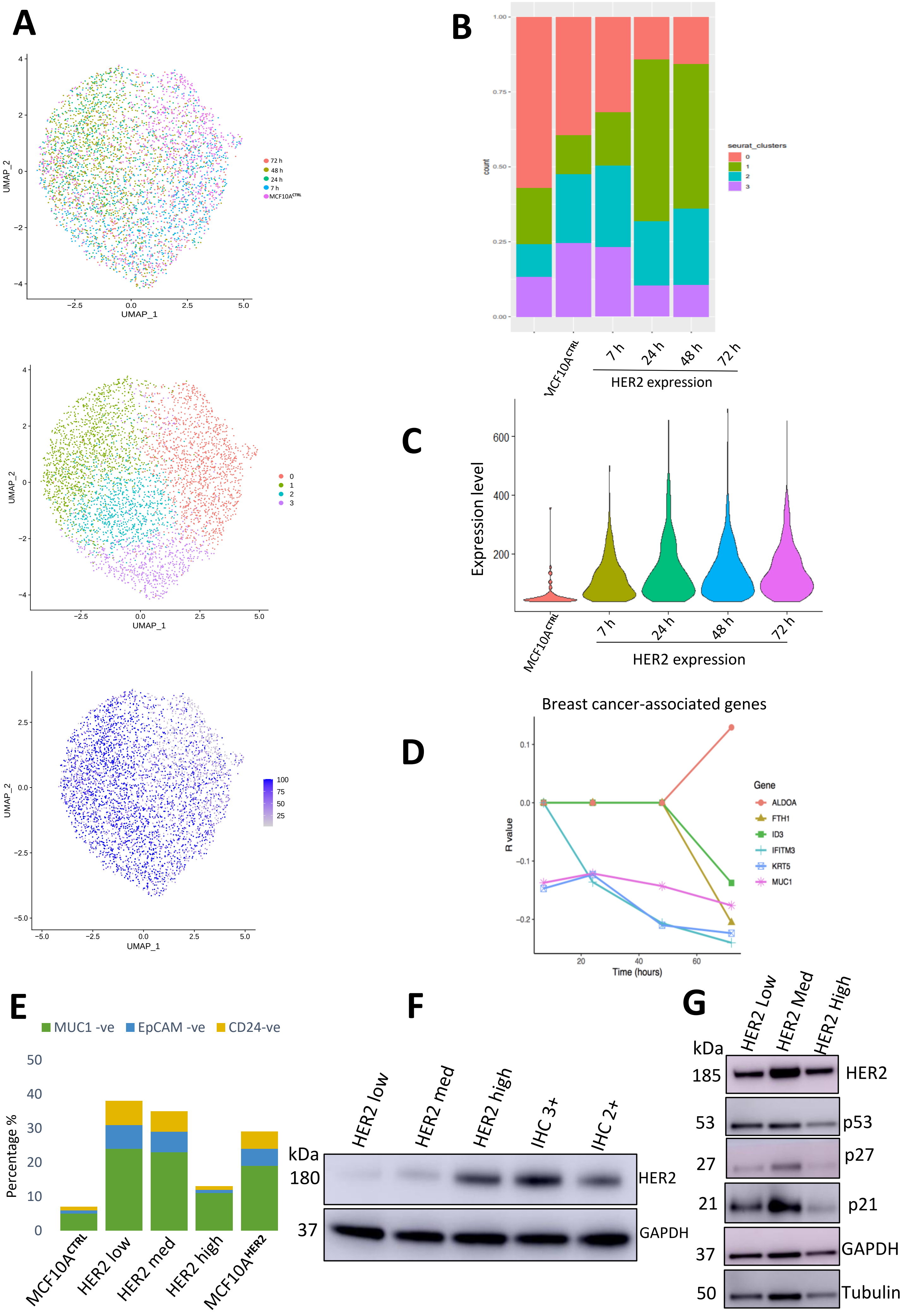

Disease Models & Mechanisms • Supplementary information

Fig. S3. Identification of novel genes associated with breast cancer.

(A) UMAP plot showing clustering based on different time points. UMAP plot displaying clusters of genes with similar features. UMAP plot showing a range of HER2 gene expression.

(B) Bar graph showing Seurat clustering which defines clustering via differential gene expression.

(C) Violin plot shows HER2 levels increase in a time-dependent manner with HER2 expression.

(D) Single cell RNA sequencing was performed on MCF10A cells with HER2 induction from 0 to 72 hours (3 days). Line graph shows R values as a measure of linear relationship between HER2 expression increase (with time) and some genes of interest that either increase in expression or decrease in with HER2 expression.

(E) Cells were analysed by flow cytometry and HER2 positive cells were separated into three subpopulations of low, medium, and high HER2 overexpression as indicated. The enrichment of stem markers is shown as a proportion of the total number of cells exhibiting MUC1 –ve, EpCAM –ve and CD24 -ve phenotype.

(F) HER2 expression between low, medium, and high HER2 expressing cells was compared with two primary human samples (IHC 3+ and IHC 2+). GAPDH was used a loading control. Panel 1 has an exposure time; 35 seconds. Panel 2 has an exposure time of 60 seconds. N=1.

(G) Western blot of the indicated proteins known to have higher expression in cells that have undergone OIS. Protein lysates were prepared from cells sorted based on HER2 expression. HER2 was induced in cells for 3 days (MCF10AHER2) and then FACS separated based on HER2 expression into three different subtypes (low, medium, and high HER2 expressing cells). GAPDH and Tubulin were used as loading controls. N=3.

Supplementary Figure 4

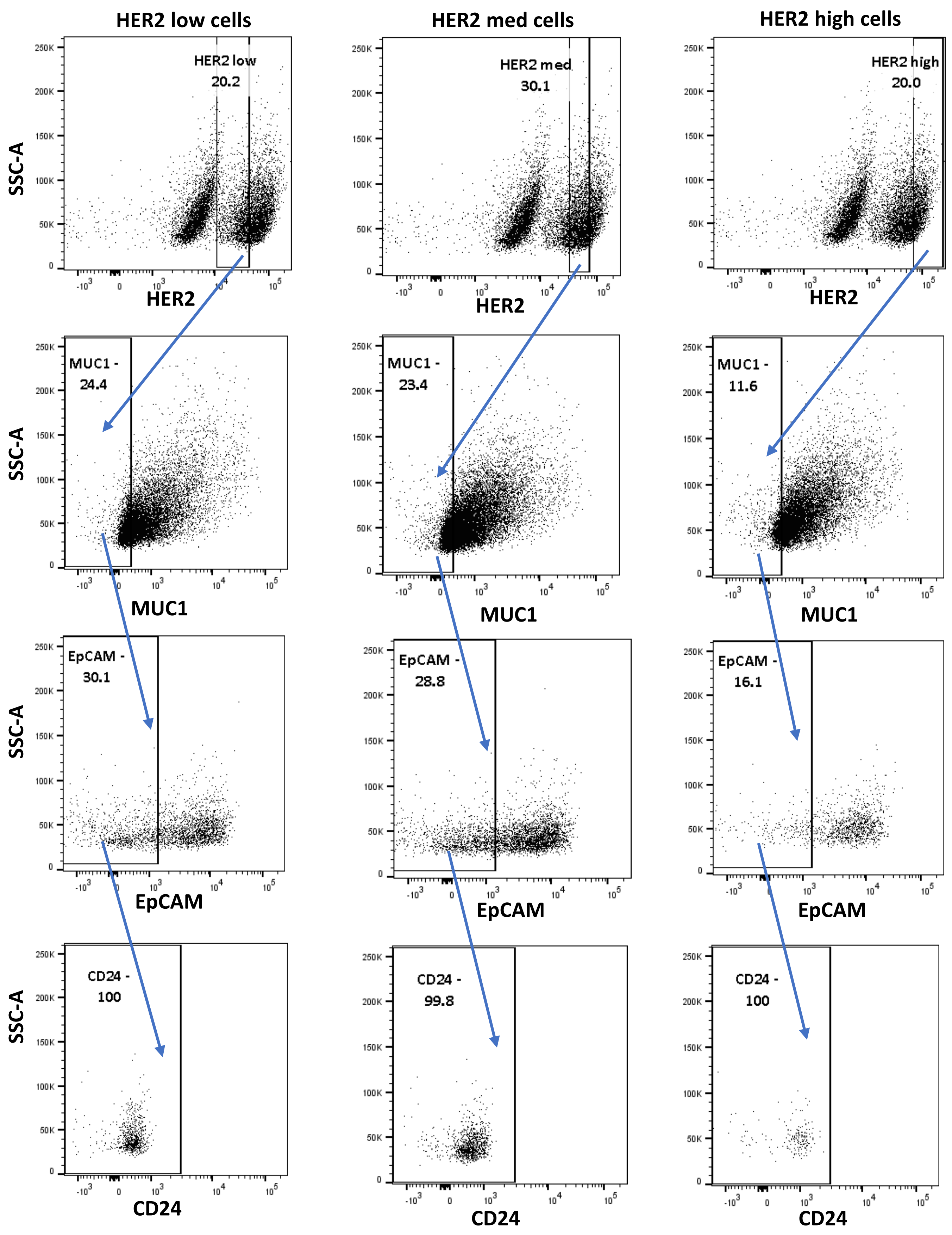

Fig. S4. Analysis pipeline of how HER2 levels were associated with breast stemness by FACS. Cells were analysed by flow cytometry and HER2 positive cells were separated into three subpopulations of low, medium, and high HER2 overexpression as indicated. The enrichment of stem markers is shown as a proportion of the total number of cells exhibiting MUC1 -ve and EpCAM -ve and CD24 -ve phenotype. The blue arrows indicate step-by-step analysis of the HER2 subpopulations, and the respective enrichment of breast stem markers in each subtype.

Dataset 1. Phosphoproteomic analysis of HER2 signalling.

Click here to download Dateset 1

Disease Models & Mechanisms • Supplementary information
